# Dynamic Response Mechanisms of Anammox Reactors Under Nitrogen-Loading Fluctuations: Nitrogen Removal Performance, Microbial Community Succession, and Metabolic Functions

**DOI:** 10.3390/microorganisms13040899

**Published:** 2025-04-14

**Authors:** Xuemei Liu, Kai Wan, Chunqiao Xiao, Jingang Hu, Xiangyi Deng, Ruan Chi

**Affiliations:** 1 Key Laboratory for Green Chemical Process of Ministry of Education, Wuhan Institute of Technology, Wuhan 430205, China; 11706060001@stu.wit.edu.cn (X.L.); q541702060@163.com (K.W.); 12116010002@stu.wit.edu.cn (J.H.); xiangyideng@126.com (X.D.); 2 School of Chemical and Materials Engineering, College of Post and Telecommunication of Wuhan Institute of Technology, Wuhan 430073, China

**Keywords:** anaerobic ammonia oxidation, rare earth tailings, microbial community structure, co-occurrence network, substrate fluctuations, metabolic pathways

## Abstract

The leachate from ion-adsorbed rare earth tailings poses challenges to the application of the anaerobic ammonium oxidation (anammox) process in this field due to its large fluctuations in ammonia nitrogen concentration (50–300 mg/L) and high flow rate (4000–10,000 m^3^/d). This study investigated the effects of nitrogen-loading rate (NLR) regulation on denitrification performance through reactor operation and elucidated the mechanisms of NLR impacts on anammox processes via microbial community analysis and metabolic profiling. The results revealed a nonlinear relationship between nitrogen loading and system performance. As NLR increased, both denitrification efficiency and anammox bacterial abundance (rising from 5.85% in phase P1 to 11.43% in P3) showed synchronous enhancement. However, excessive nitrogen loading (>3.68 kg/m^3^·d) or nitrogen starvation led to performance deterioration and reduced anammox bacterial abundance. Microbial communities adopted modular collaboration to counteract loading stress, with modularity indices of 0.563 and 0.545 observed in the inhibition phase (P2) and starvation phase (P4), respectively. Zi-Pi plot analysis demonstrated a significant increase in inter-module connectivity, indicating reinforced interspecies interactions among microorganisms to resist nitrogen-loading fluctuations.

## 1. Introduction

Ionic rare earth ore is a kind of important mineral resource that is rich in medium and heavy rare earths with the advantages of low radioactivity ratio and high economic value, which are of great strategic significance to the development of high-tech fields such as electronic products, superconducting materials, precision aerospace materials, and so on. Based on the mineral endowment characteristics of the in situ leaching process developed due to the advantages of a smaller mine breakage, high recovery rate of rare earths, and lower mining costs, ionic rare earth ores in 80~90% of the rare earth elements in the trivalent cation state adsorbed in kaolin, eolite, and other clay minerals have now become mainstream in the industrialization of the ionic rare earth deposit mining method [1,2,3]. At present, 0.5–2% (NH_4_)_2_SO_4_ is usually used as a leaching agent to desorb rare earth elements into the rare earth mother liquor; however, the application of the in situ leaching process requires the consumption of 7–12 tons of ammonium sulfate for each ton of rare earth ore leached, of which about 10–30% of ammonium salts will be retained in the mine soil [4], which will result in ammonia nitrogen concentrations that are 12–40 times higher than those of the original soil prior to mining, and the H^+^ generated by hydrolysis of residual ammonia nitrogen in the soil will lead to acidification of the soil and affect the growth of vegetation in the mining area [5]. Under the influence of rainfall leaching, the residual ammonium salts will be leached out of the mine into the surrounding water and soil, bringing continuous harm to the surrounding environment [6]. The ion-adsorbed rare earth tailings leachate exhibits seasonal variations, characterized by high flow rate fluctuations (4000–10,000 m^3^/d), wide ammonia nitrogen concentration ranges (50–300 mg/L), and low organic matter content (COD < 10 mg/L) [7]. As the primary contaminant in rare earth tailings leachate, ammonia nitrogen removal has become a critical environmental issue requiring immediate resolution.

Biological denitrification has been an important tool for ammonia nitrogen wastewater treatment. Traditionally, the biological nitrification/denitrification (N&DN) process is widely used to remove nitrogen from wastewater. In this process, ammonia nitrogen is first oxidized to nitrate by nitrifying bacteria under aerobic conditions, and then denitrifying bacteria by converting nitrate to nitrogen under anoxic conditions using organic carbon as an electron donor. However, this process requires additional aeration in the nitrification stage to promote the conversion of ammonia and nitrogen and requires a large amount of organic carbon to be added externally in the denitrification stage to meet the demand for denitrification and ensure the rate of nitrogen removal [8]. However, the low content of organic matter as well as dissolved oxygen in the leachate of rare earth tailings makes it difficult to apply the traditional nitrification/denitrification technology [9]. Anaerobic ammonia-oxidizing bacteria (AnAOB) are able to utilize nitrite as an electron acceptor to convert ammonia nitrogen to nitrogen gas under anoxic conditions [10]. Therefore, the partial nitrification–anaerobic ammonia oxidation (PN/A) process is able to reduce the aeration energy consumption by 60%, sludge formation by 90%, and organic carbon source by 100% compared with the traditional nitrification/denitrification process, which greatly reduces the cost of ammonia nitrogen wastewater treatment [11,12]. Anaerobic ammonia oxidation has been widely studied in the treatment of high ammonia nitrogen and low carbon-to-nitrogen ratio wastewater generated from chemical, coking, pharmaceutical, and rubber industries [13,14,15]; however, anaerobic ammonia-oxidizing bacteria (AnAOB), which are the core of the process, are very sensitive to the environment, and their activity is easily reduced by the interference of other factors such as heavy metals, antibiotics, and nanoparticles in wastewater [16,17,18,19].

Notably, changes in nitrogen loading also affect the activity of AnAOB, with lower nitrogen loading affecting the substrate mass transfer process, decreasing the metabolic activity of AnAOB, and inducing the initiation of programmed cell death in bacteria, which reduces the number of viable bacteria [20,21]. And when the substrate concentration is too high, the residual ammonia nitrogen and nitrite will be converted into free nitrite (FNA) and free ammonia (FA), which are more likely to diffuse into the cell through the cellular lipid membranes, and the changes in the intracellular levels of FA and FNA will change the intracellular pH, causing changes in the cellular transmembrane potential and leading to cell lysis and death [22,23,24]. Although anaerobic ammonia-oxidizing granular sludge secretes large amounts of extracellular polymers to resist the inhibitory effect of high nitrogen loading, this can lead to blockage of gas release channels in anaerobic ammonia-oxidizing granular sludge, causing particles to float upward and increasing the risk of loss of anaerobic ammonia-oxidizing granular sludge [25,26,27]. Dramatic fluctuations of ammonia and nitrogen concentrations in rare earth tailings leachate pose an obstacle to the stable operation of the anaerobic ammonia oxidation process in rare earth tailings wastewater.

Therefore, in order to promote the industrial application of the anaerobic ammonia oxidation process in rare earth wastewater effluents, the present study was conducted to investigate (1) the response of the denitrification performance of the anammox reactor to the fluctuation of the nitrogen load by intermittently modulating the nitrogen load change in the reaction system, (2) resolving the change in the structure of the microbial community under the fluctuation of the nitrogen load, (3) based on the KEGG metabolic pathway database, the use of the PICRUSt2 for functional annotation and prediction of 16S rRNA amplicon data to analyze the abundance changes of metabolic pathways in microbial communities at different nitrogen-loading stages. To advance the industrial implementation of the anammox process for treating rare earth mining wastewater, this study aims to deliver robust empirical data and a theoretical framework to guide practical applications.

## 2. Materials and Methods

### 2.1. Anammox Reactor Configuration

**Figure 1:**
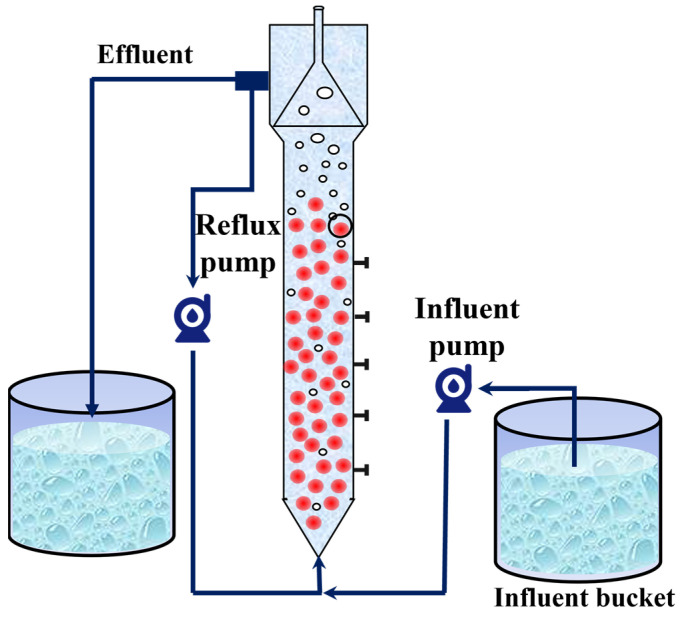
Schematic diagram of anammox reactor.

In this study, an expanded granular sludge bed (EGSB) with a volume of 10 L was used as an anammox bioreactor. The reactor was made of Plexiglas and covered with insulating foam for warmth and protection from light. The reactor was equipped with inlet and return pipes, and a peristaltic pump was used to realize the continuous inlet and return of synthetic wastewater (Figure 1). The seed sludge was obtained from Jiangsu University of Science and Technology (JUST), and the nitrogen-loading rate (NLR) and nitrogen removal rate (NRR) of the anammox reactor were maintained at 1.38 ± 0.01 kg/m^3^·d and 1.15 ± 0.02 kg/m^3^·d, respectively, after 15 days of laboratory operation.

### 2.2. Synthetic Wastewater and Operational Strategy

The required NH_4_^+^-N and NO_2_^−^-N in the synthesized wastewater were provided by (NH_4_)_2_SO_4_ and NaNO_2_, respectively. Based on the anammox reaction stoichiometry ratio, the NH_4_^+^: NO_2_^−^ of the influent water was controlled to be 1:1.32 [28]. Table 1 shows the details of the synthetic wastewater.

The whole experimental process was divided into 5 stages according to the nitrogen-loading concentration. Phase 1 (1–15 days) served as the control group for the whole experiment, and the nitrogen loading was stably maintained at 1.38 ± 0.01 kg/m^3^-d, and the nitrogen concentration of each type of nitrogen in the effluent water was detected every day as the basic data, which was used for comparison with the subsequent stages of nitrogen-loading change. In phase 2 (16–65 d), the tolerance capacity of anammox was explored by using 30 mg/L of NH_4_^+^-N as a concentration gradient and adding NH_4_^+^-N and NO_2_^−^-N proportionally, during which the substrate concentration was gradually increased. In phase 3 (days 66–105), the effect of low nitrogen-loading conditions on the denitrification ability of anammox was explored by rapidly decreasing the substrate concentration, and the recovery was examined by gradually increasing the substrate concentration. In phase 4 (106–120 d), the nitrogen removal performance of an anammox reactor under starvation conditions was explored by decreasing the substrate concentration again and stopping the water intake. In phase 5 (121–145 d), the recovery period, the substrate concentration was gradually increased again to investigate the recovery of the reactor after the starvation stage. The pH of the influent water to the reactor was controlled to be 7.30 ± 0.50, the hydraulic retention time to be 3.6 h, and the temperature to be 30.0 ± 2.0 °C throughout the experiment.

### 2.3. Analytical Methods

The influent and effluent samples were collected and filtered through a 0.45 µm needle filter. The concentrations of NH_4+_-N, NO_2_^−^-N, and NO_3_^−^-N in the water samples were determined using Nessler’s reagent, N(1-naphthyl) ethylenediamine dihydrochloride, and UV spectrophotometry (Shimadzu, Kyoto, Japan) according to the standard method, respectively [29]. Performance metrics of the anammox bioreactor, such as total nitrogen removal (NRE), nitrite removal (NR), ammonia nitrogen removal (ANR), and NRR, were calculated according to the methodology described by Wang et al. [30]. Free ammonia (FA) and free nitrite (FNA) were calculated according to the method provided by Wu et al. [28]. The ratio of anammox and denitrification pathways in contributing to total inorganic nitrogen (TN) removal was calculated using Equations (1) and (2), respectively, according to the method provided by Ji et al. [31]. Reactor pH and temperature were monitored using a Multi 3620 IDS (WTW, Wilhelm, Germany). Origin 2021 software was used to visualize and analyze data from the daily operation of the anammox bioreactor.

(1) $$\mathrm{Anammox}\;\left(\%\right)=([{\mathrm{NH}}_{4}^{+}\text{-}N{]}_{\inf}-[{\mathrm{NH}}_{4}^{+}{\text{-}N]}_{\mathrm{eff}})\times (1+1.32-0.26)/([TN\text{-}\inf]-[TN\text{-}\mathrm{eff}])$$

(2) Denitrification (%)=100−Anammox contribution

### 2.4. High-Throughput Sequencing

Five parallel sludge samples were collected at the end of each phase to assess changes in microbial community abundance under the influence of substrate fluctuations in each phase, using the E.Z.N.A. Soil DNA kit (Omega Bio-Tek, Doraville, GA, USA) to extract DNA from each sludge sample. Qualified DNA was analyzed on the MiSeq platform (Illumina, Inc., San Diego, CA, USA) and sequenced by Shanghai Majorbio Bio-Pharm Technology Ltd. (Shanghai, China). PCR amplification of the highly variable region V3-V4 of the bacterial 16S rRNA gene was performed using 341F (5′-CCTACGGGGNGGCWGCAG-3′) and 806R (5′-GGACTACHVGGGTWTCTAAT-3′) as primers. The amplified products were purified and subjected to 2 × 300 bp bipartite sequencing on the Illumina MiSeq platform, and the raw data were processed by the QIIME2 process, including quality filtering (quality threshold Q20), chimera removal (UCHIME algorithm), OTU clustering (97% similarity threshold), and ultimately, species annotation through the Silva 138 database.

## 3. Results and Discussion

### 3.1. Performance Variation of Anammox Bioreactor

**Figure 2:**
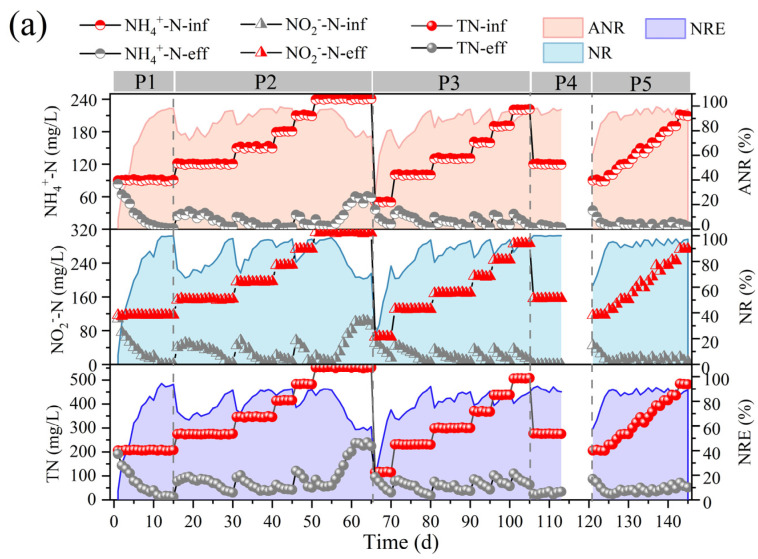

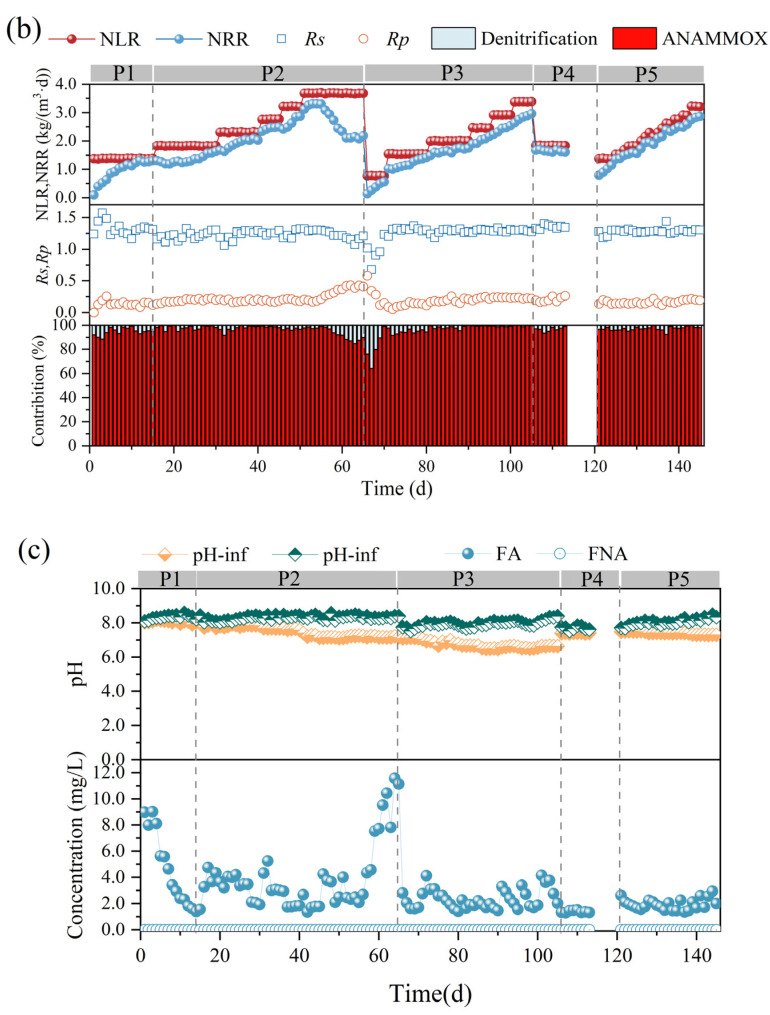
Performance variations of anammox bioreactor at different stages (P1–P5). (**a**) Ammonia nitrogen removal (ANR), nitrite removal (NR), total nitrogen (TN), and total nitrogen removal efficiency (NRE). (**b**) Changes in nitrogen removal rate (NRR), nitrogen-loading rate (NLR), stoichiometric ratios (*Rs* and *Rp*), and nitrogen removal contribution. (**c**) Changes in reactor pH and free ammonia (FA) and free nitrite (FNA) content.

The effect of nitrogen-loading changes on the denitrification performance of the reactor was investigated by testing the effluent nitrogen content of the anammox bioreactor at various stages, and the changes in the indicators of the anammox reactor under different nitrogen-loading conditions are demonstrated in Figure 2.

In the first phase, the nitrogen-loading rate (NLR) and nitrogen removal rate (NRR) of the anammox reactor reached 1.38 ± 0.01 kg/m^3^·d and 1.15 ± 0.02 kg/m^3^·d, respectively, on the basis of a long-term stabilized operation in the laboratory (Figure 2b). The ANR, NR, and NRE of the reactor were maintained at 96.07 ± 0.93 mg/L, 94.80 ± 1.46 mg/L, and 83.47 ± 1.24 mg/L, respectively (Figure 2a), and the stoichiometric ratios, *Rs* (∆NO_2_^−^-N/∆NH_4_^+^-N) and *Rp* (∆NO_3_^−^-N/∆NH_4_^+^-N), were 1.27 ± 0.03 and 0.28 ± 0.02, respectively (Figure 2b), which were close to the theoretical values of anammox (*Rs* = 1.32, *Rp* = 0.26), indicating that anammox is the main denitrification pathway at present, whereas the deviation of the reaction stoichiometry ratio from the theoretical values may be caused by the decrease in NO_3_^−^-N as well as NO_2_^−^-N due to the side reactions, such as denitrification, in the reactor, which is also consistent with the contribution of denitrification in Figure 2c.

In the second phase (16–65 d), with a concentration gradient of 30 mg/L ammonia nitrogen and proportional addition of ammonia nitrogen and nitrite nitrogen, a small decrease in ANR, NR, and NRE was observed at the initial stage of substrate concentration increase, but after acclimatization, a recovery of the performance was achieved, which led to a gradual increase in the NRR of the reactor from 1.15 ± 0.02 kg/m^3^·d eventually to 3.26 ± 0.07 kg/m^3^·d. It was shown that an appropriate increase in ammonia nitrogen could promote the level of nitrogen removal [27]. However, when the NH_4_^+^-N and NO_2_^−^-N concentrations were continuously maintained at 240.45 ± 0.44 mg/L and 311.32 ± 1.00 mg/L, at which time the NLR reached 3.68 ± 0.01 kg/m^3^·d, the ANR and NR decreased from 96.40 ± 2.06% and 96.57 ± 1.67%, respectively, to 76.97 ± 2.34% and 68.58 ± 2.57%, and the NRR decreased to 2.13 ± 0.04 kg/m^3^·d, which indicated that the high nitrogen loading in the reactor exceeded the tolerance capacity of the reactor, and the anammox was inhibited, resulting in further deviation of *Rs* and *Rp* from the theoretical values. At this time, FA was elevated to 9.41 ± 1.49 mg/L (Figure 2c). It has been reported that high concentrations of FA can lead to inhibition of microbial metabolic activity by disrupting the transmembrane proton gradient and causing proton imbalance [32], and inhibition of nitrifying bacteria (NOB) was observed at 0.1–1.0 mg/L, while inhibition of ammonia-oxidizing bacteria (AOB) was observed at 10 mg/L [33]. Fernández et al. found that anammox performance became highly unstable and removal efficiency decreased at FA concentrations of 20–25 mg/L [34], and Waki et al. reported that anaerobic ammonia-oxidizing bacteria (AOB) may be inhibited at FA levels of 13–90 mg/L [35], and the increase in FA concentration is one of the reasons for the overall decrease in NRR. The lower tolerance limit of FA in the anaerobic ammonia oxidation process in this study was lower than that in previous studies, and the differences between the results may be attributed to the differences in operating conditions, physical structure of sludge (flocculated sludge, biofilm, or granular sludge), and microbial populations [13,22]. It is noteworthy that AOB showed higher tolerance and recovery rate when inhibited by FA [33], so the decrease in Rs values and increase in Rp values could be attributed to the enhanced ammonia oxidation reaction in the reactor, which promotes the conversion of ammonia nitrogen to nitrate in the reactor.

In the third phase (66–105 d), the influent ammonia nitrogen as well as nitrite nitrogen concentrations were reduced to 50.46 ± 0.60 mg/L and 65.60 ± 0.54 mg/L, respectively, to investigate the changes in the denitrification performance of the anammox reactor under low nitrogen-loading conditions. Low substrate concentration can dilute the remaining high substrate concentration in the reactor during P3 and help to eliminate the inhibition of the reactor by high nitrogen loading [24]. However, low substrate concentration in the feed water not only leads to a decrease in the mass transfer efficiency of the substrate, but also significantly alters the osmotic pressure and redox potential of the reactor, which can easily lead to the hydrolysis of biomass [23]. Moreover, the activity of AnAOB was at a low level due to the long-term inhibition by the high concentration of substrate, so ANR, NR, and NRE did not immediately recover to the pre-inhibition level at the beginning of P3. However, with continuous operation under low nitrogen-loading conditions, the performance of the anammox reaction period was effectively restored, and ANR, NR, and NRE gradually increased, NRR was effectively improved, and RS and RP gradually approached the theoretical values.

In the fourth phase (105–120 d), a starvation phase, the effect of the rare earth tailings dry period on the anammox reactor was simulated by decreasing the substrate concentration and stopping the water intake. The reactor performance did not show significant fluctuations when ammonia nitrogen and nitrite nitrogen decreased to 120.32 ± 0.55 mg/L and 156.08 ± 0.20 mg/L, respectively, and then, water intake was stopped for one week to starve the reactor. In phase 5, the influent ammonia nitrogen and nitrite nitrogen concentrations were set to 90 mg/L and 120 mg/L, respectively, and the reactor was restarted. Although the nitrogen removal efficiency was low in the initial recovery period, the reactor ANR as well as NE reached more than 94% after one week of operation, and then, the nitrogen removal performance recovered rapidly along with the stage-by-stage increase in nitrogen loading, and the NRR finally reached 2.71 ± 0.15 kg/m^3^·d.

AnAOB are environmentally sensitive, and their activity is susceptible to interference by heavy metals, antibiotics, and organics in wastewater in addition to nitrogen loading during the operation of the anaerobic ammonia oxidation process. Su et al. (2021) reported that the blockage of key metabolic pathways of AnAOB by the rare earth ion La(III) may be the main manifestation of La(III) toxicity [16], whereas Cd(II) was able to enter the biofilm to affect the activity of anammox [36], and phenol was able to lead to the decrease in the abundance of AnAOB and promote the proliferation of denitrifying bacteria Azoarcus and Thauera that degrade phenol. Phenol, 2-nitrophenol, and 4-nitrophenol can bind to nitrite reductase, preventing the first step of the anammox reaction and inhibiting the denitrification performance of the anammox process [14]. In order to maintain the stability of anammox and nitrogen removal performance as well as rapid recovery, it is often necessary to regulate physical and chemical parameters such as reactor temperature, pH, and temperature to increase the resistance of the anammox process to unfavorable factors and to promote the recovery of performance. Control of pH and temperature is critical for anammox performance and prevention of substrate inhibition [37].

### 3.2. Evolution of Microbial Communities in Bioreactors Under the Influence of Nitrogen Loading

In order to deeply analyze the effect of nitrogen-loading changes on the microbial community, sludge samples were collected at the end of each stage to analyze the microbial community composition by high-throughput sequencing. The alpha diversity indices of bacteria in each stage of the reactor are shown in Table 2. Good’s coverage ensured the reliability of this sequencing, indicating that the vast majority of species in the anammox reactor were detected [16]. In P2 and P3 phases, along with the continuous increase in nitrogen loading, Shannon diversity index and ACE richness decreased compared to the P1 phase, and the decrease in Pielou evenness index also indicated the decrease in species evenness in the community, which may be because the high-nitrogen-loading-tolerant species became the dominant species and seized the ecological niches, which led to a decrease in microbial diversity and richness in the reactors [38,39]. However, during P4, the dominant microorganisms were suppressed as nitrogen loading was reduced and starvation took hold, leading to opportunistic population accretion, resulting in a rebound in the diversity index and an upward increase in the Pielou evenness index.

**Figure 3:**
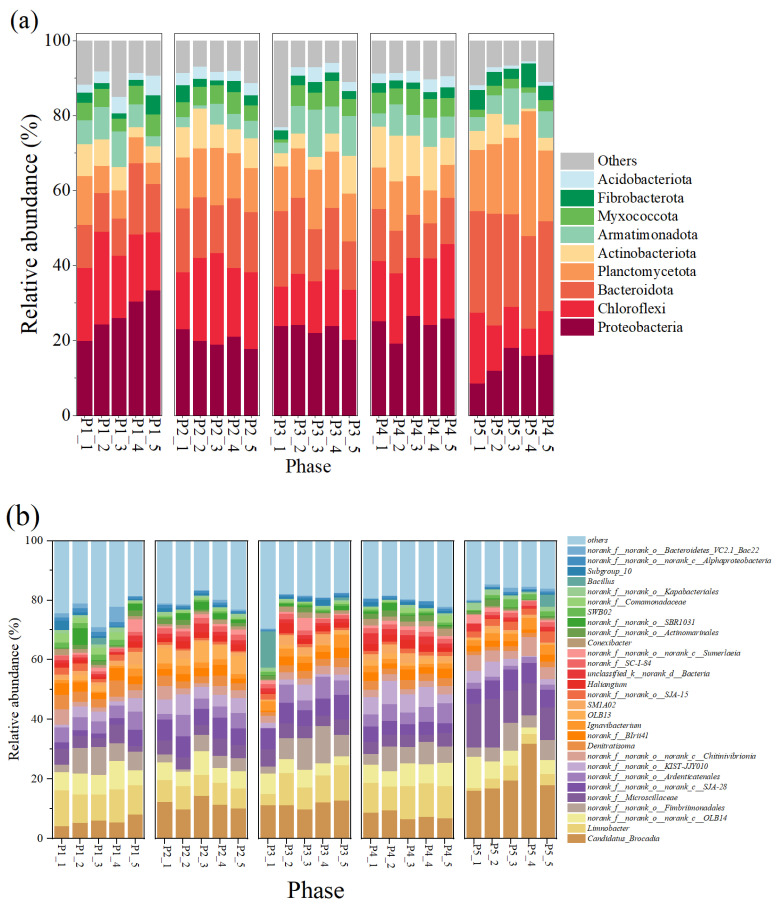
Composition of microorganisms and changes in their relative abundance in the reactor. (**a**) Phylum level. (**b**) Genus level.

At the phylum level (Figure 3a), Proteobacteria, Chloroflexi, and Bacteroidota and Planctomycetes, as the major bacterial phyla in this reactor, maintained high abundance in all phases, respectively, ranging from 14.16 ± 3.40% to 26.82 ± 4.74%, 12.18 ± 3.73 to 20.10 ± 3.18%, 12.72 ± 3.28 to 26.06 ± 2.15%, and 8.11 ± 2.58 to 21.50 ± 6.02%, respectively. Among them, Proteobacteria contain many nitrifying and denitrifying functional bacteria that are widely present in nitrogenous wastewater, and their presence is important for nitrogen metabolism in the reactor [16,40]; members of Chloroflexi contain numerous filamentous bacteria that are commonly found in treatment plants with a primary focus on nitrogen and phosphorus removal [41]. AnAOB were the main component of Planctomycetes, which indirectly suggests an increase in the abundance of AnAOB, which is consistent with the results of enhanced nitrogen removal from anammox reactors.

The changes in microbial community composition at the genus level were further analyzed (Figure 3b). *Candidatus brocadia* was the predominant AnAOB in the reactor; its relative abundance was 5.85 ± 1.30% at the P1 stage, and as the nitrogen loading was increased, its relative abundance reached 11.65 ± 1.67% and 11.43 ± 1.02% at P2 and P3, respectively (Figure 3b), and 1.02% at P2 and P3, respectively (Figure 3b). This suggests that appropriately high nitrogen loading enriches AnAOB and improves reactor performance, which is also echoed by the increased denitrification performance of the reactor in Figure 2. In addition, some heterotrophic bacteria such as *OLB13*, *OLB14*, *Denitratisoma*, *Limnobacter*, and *SJA-28* showed high abundance in the reactor. Among them, *OLB13* and *OLB14* are members of the Chloroflexi phylum, which suggests that their presence promotes the metabolism of cellular debris and extracellular proteins in the reactor and promotes bacterial aggregation [41,42]. In addition, *OLB13* possesses denitrification ability with *Denitratisoma*, *Ignavibacterium*, and *SJA-28*, which can promote the accumulation of NO_2_^−^-N to provide a reaction substrate for anammox and contribute to the enhancement of NRE [43,44,45]. The presence of these dominant microorganisms enhanced denitrification, which may also explain the deviation of reactor *Rs*, *Rp* from the theoretical values for anammox reactions.

**Figure 4:**
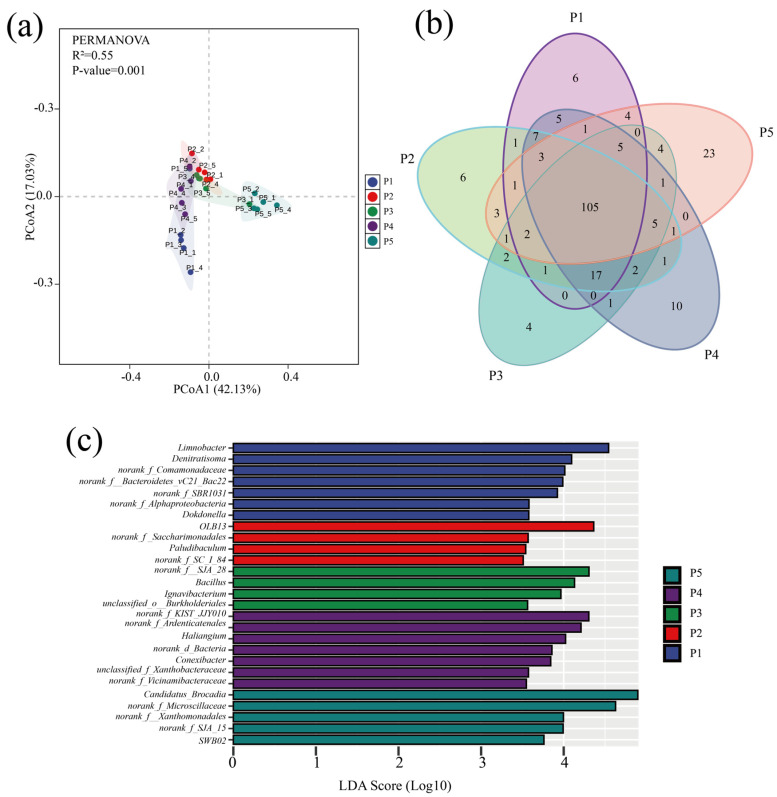
Analysis of microbial community differences. (**a**) PcoA analysis of microbial communities at different stages. (**b**) Venn diagram of genus-level distribution of microbes in samples at different stages (colors represent samples at different stages, numbers represent species contained in the samples). (**c**) LEfSe analysis of genus-level microbial communities in different run stages (LDA scores > 3.5).

Variability in microbial community structure at different stages of the anammox reactor was analyzed by principal coordinate analysis (PCoA) based on the Bray–Curtis distance matrix, and the significance of the differences was assessed by combining multivariate permutation analysis of variance. As shown in Figure 4a, the PCoA analysis results explained 59.16% of the community variability, and the multiple replacement variance results indicated that the microbial communities were significantly different (*p* < 0.001) overall. Despite the significant differences in the microbial community road clusters at each stage, the Venn plot showed (Figure 4b) that a total of 105 species were found in the samples at all phases, suggesting that the variability in the community results was mainly due to variations in the abundance of species.

Genera with significant abundance differences in the five stages were compared by LEfSe analysis (*p* < 0.05) (Figure 4c). The results indicated that the dominant bacteria such as *Denitratisoma*, *Limnobacter*, *OLB13*, *SJA-28*, and *Candidatus brocadia* were the indicator microorganisms with the largest relative abundance changes (LDA > 4) at each phase; the dominant microorganisms with high abundance had a driving role in the evolution of microbial community structure, and their relative abundance change is an important indicator to respond to the differences in bacterial and fungal community structure across regions [46].

**Figure 5:**
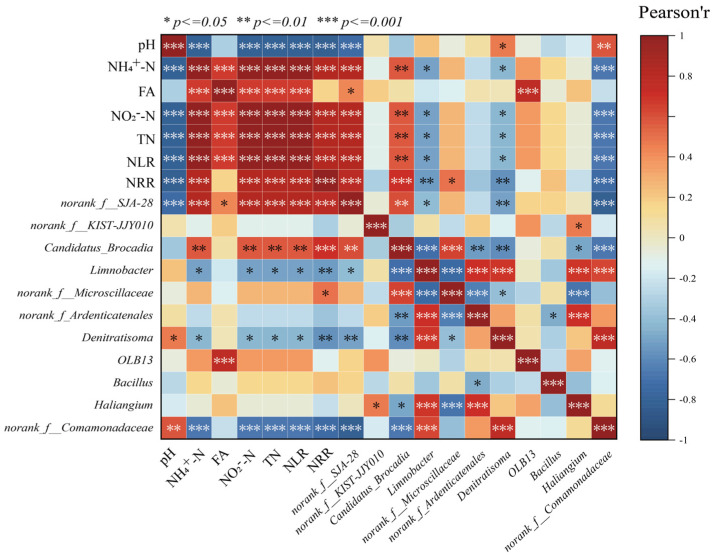
Correlation analysis of physicochemical properties with microorganisms.

The correlations between environmental factors and significantly different microbial species in the LEfSe analysis were revealed by correlation heatmaps (Figure 5). Among them, NH_4_^+^-N, NO_2_^−^-N, TN, and NLR showed significant positive correlation (*p* < 0.001) with *Candidatus brocadia*, indicating that AnAOB can be enriched under the appropriate substrate concentration conditions, which can further increase the nitrogen removal rate of anaerobic ammonia-oxidizing reactors, and thus, *Candidatus brocadia* and NRR showed a significant positive correlation. In addition to the influence of physicochemical factors on the abundance of *Candidatus brocadia*, the competition between species should not be ignored; the reaction stoichiometry ratio consistently deviated from the theoretical value of anammox suggesting that there were other reactions involved in the de-nitrogenation process in the reactor, while *Candidatus brocadia* was significantly negatively correlated (*p* < 0.001) with the related microorganisms with denitrifying function, such as *Denitratisoma*, *Limnobacter*, etc. This phenomenon may result from partial mortality of AnAOB and other microorganisms under nitrogen-loading inhibition. The dead cells served as carbon sources, providing nutrients for denitrifying bacteria (e.g., Denitratisoma and *Limnobacter*), thereby increasing their relative abundance during the later phase of P2. Consequently, the contribution of denitrification to nitrogen removal was enhanced. However, during P3 and P5, despite continuous increases in nitrogen loading, the threshold tolerance of AnAOB was not exceeded. This reduced the inhibition-induced mortality of AnAOB under high nitrogen loads. Simultaneously, the decreased availability of carbon sources limited the metabolic activity of heterotrophic bacteria such as *Denitratisoma* and *Limnobacter*, leading to a decline in their relative abundance. Therefore, NH_4_^+^-N, NO_2_^−^-N, TN concentrations, and NLR exhibited negative correlations with the relative abundances of *Denitratisoma* and *Limnobacter*.

### 3.3. Co-Occurrence Network Analysis

Microbial co-occurrence networks are an important tool for describing interactions within microbial communities, and there is growing evidence that the distribution of co-occurrence networks is characterized by feedback from microorganisms in the community in response to changes in the environment [46]. The response of the microbial community inside the anammox reactor to changes in nitrogen loading was explored through a SparCC co-occurrence network. Nodes in the network denote various microbial genera, while colors indicate the phylum to which they belong, and significantly strong correlations with SparCC correlation coefficients |r| > 0.8 and significance *p* < 0.05 between species were calculated and retained as edges of the network (Figure 4). Among them, the number of positively correlated edges of the co-occurrence network was maintained at 52.78% to 57.05% (Table 3), indicating that microorganisms have extensive synergistic symbiotic relationships, and the topologically, the microbial co-occurrence networks in all stages except P3 had a significant modular structure (modularity > 0.4) (Table 3), suggesting that microorganisms with similar metabolic functions or possessing synergistic symbiotic relationships are closely related [47]. During the low nitrogen-loading phases (P1 and P4), despite the high nitrogen removal performance of the reactor, the microbial diversity remained elevated due to the absence of nitrogen-induced inhibition. In contrast, the excessive nitrogen load in phase P2 imposed nitrogen stress on the microbial community, resulting in cell lysis and mortality [32]. The demise of low-tolerance microorganisms provided carbon sources and nutrients for heterotrophic denitrifiers, leading to increased relative abundances of Denitratisoma and Limnobacter and enhanced denitrification activity. Consequently, Zi-Pi co-occurrence network analysis revealed a higher number of keystone taxa connecting modules and elevated modularity indices in phases P1, P2, and P4 compared to P3 and P5. These patterns suggest that microbial communities strengthen interspecies interactions and inter-module communication to maintain system stability under extreme nitrogen-loading conditions [46,48]. In phases P3 and P5, where nitrogen loads remained below the tolerance threshold of anaerobic ammonium-oxidizing bacteria (AnAOB), *Candidatus brocadia* dominated as the keystone species, resulting in reduced modularity and fewer inter-module connectors.

### 3.4. Metabolic Pathway Analysis of Microorganisms in the Reactor

**Figure 6:**
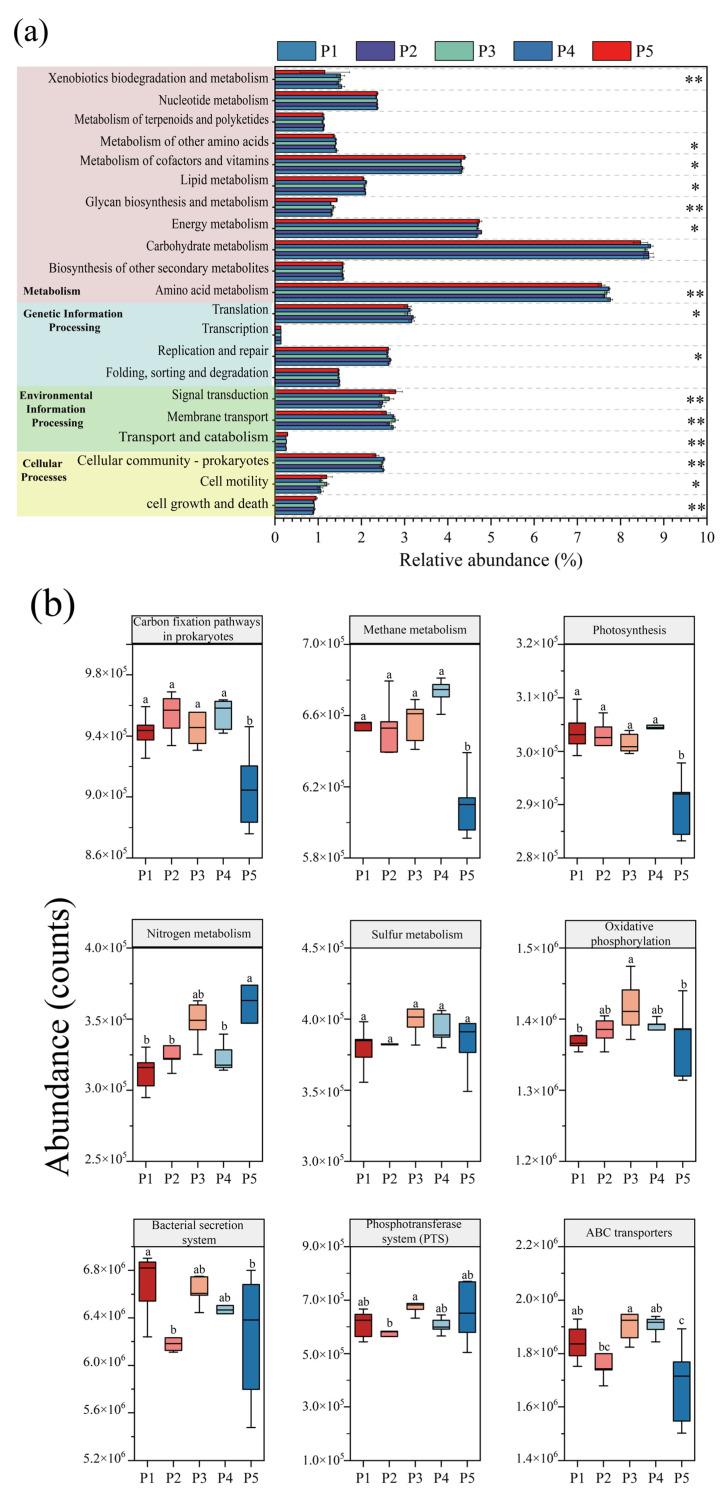
PICRUSt2 analysis of changes in microbial KEGG function during reactor operation. (**a**) KEGG secondary metabolic pathway (Kruskal–Wallis test, * *p* < 0.05, ** *p* < 0.01). (**b**) Key tertiary metabolic pathways for energy metabolism and membrane transport (Different letters indicate significant differences before and after composting.).

**Figure 7:**
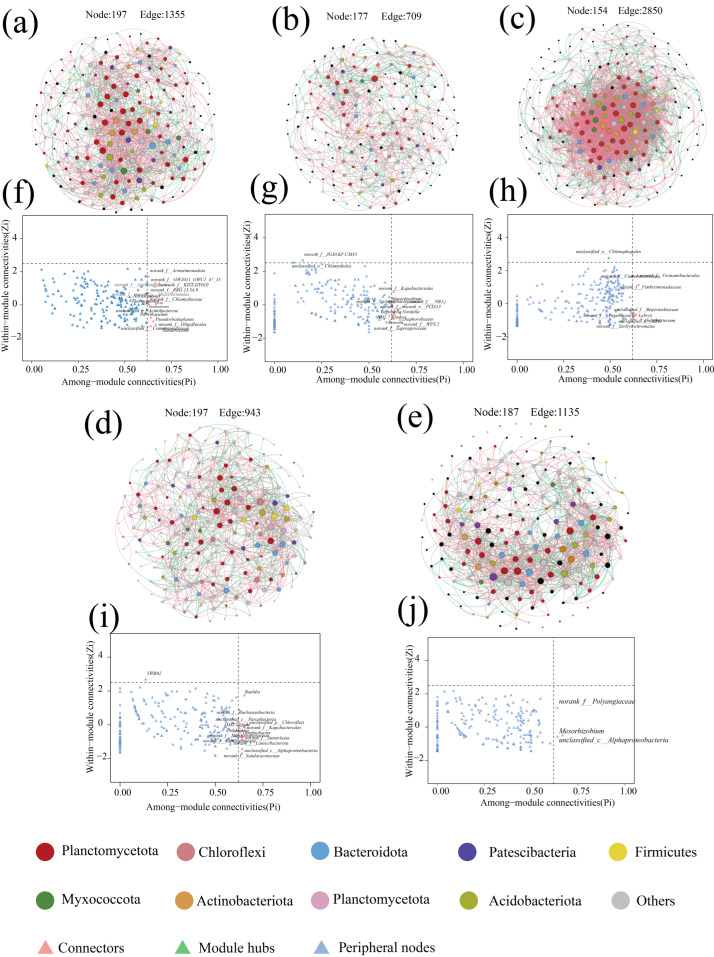
Microbial co-occurrence network analysis with network connectivity analysis. (**a**–**e**) Network diagram of microbial contribution at P1–P5 stages. (**f**–**j**) show the results of co-occurrence network connectivity for stages P1–P5. Calculated according to the SparCC correlation algorithm, the red edges between nodes correspond to a significant positive correlation (r > 0.8, *p* < 0.05), while the green edges correspond to a significant negative correlation (r < −0.8, *p* < 0.05). Node size was correlated with connectivity between microbes. zi-Pi plots show key node microbes in the co-occurrence network.

Based on the KEGG metabolic pathway database, the abundance changes in microbial metabolic pathways at different nitrogen-loading stages were systematically analyzed by using PICRUSt2 to functionally annotate and predict the data of 16S rRNA amplified fragments, and the differences in KEGG secondary metabolic pathways at each nitrogen-loading impact stage were judged by the Kruskal–Wallis test. Figure 6a shows that the pathways of “Folding, sorting and degradation”, “Transcription”, and “Nucleotide metabolism” involving genetic information processes did not change significantly, and the stability of these pathways indicates that the anammox reactor maintained the homeostasis of the underlying genetic information flow in the face of changes in nitrogen loading, ensuring the balance of gene expression, protein synthesis, and degradation. However, significant changes in other metabolic pathways suggest that microbes may maintain normal genetic proliferation by modulating other pathways in response to the effects of nitrogen loading. “Membrane transport” and “Signal transduction” are important interaction pathways between microorganisms and the environment, which are closely related to the processes of nutrient uptake, electron transfer, metabolite release, and heavy metal efflux and play a key role in microbial survival [49]. The abundance of microbial “Membrane transport” metabolic pathways in the reactor decreased significantly during the P2 and P5 phases, which may be due to the fact that at higher substrate concentrations, the nitrogen source is able to enter the microorganisms for metabolism through osmotic pressure, and the microorganisms are able to obtain nutrients more easily, resulting in fewer genes related to membrane transport, whereas at lower substrate concentrations, the transport of substrate is limited [20,21]. Therefore, the tertiary metabolic pathways regarding the “Membrane transport” system (Figure 6b) were differentially reduced at P2 as well as at P4 for “ABC transporters”, “Bacterial secretion system” and “Phosphotransferase system (PTS)”. In the “Cellular processes” module, the “Cellular communication—prokaryotes” increased under low nitrogen-loading conditions in P1 and P4, indicating an increase in communication between microorganisms, which was also consistent with the increase in the number of inter-module junctions of the co-occurrence network in P1 and P4 (Figure 7), indicating an increase in microbial connectivity. This is also consistent with the increase in the number of connections between modules of the co-occurrence network at P1 and P4 (Figure 7), and the abundance of genes of the “Cell motility” pathway increased significantly at P3 and P5, which confirms that the appropriate substrate concentration can effectively promote microbial aggregation in the reactor.

The “Metabolism” module is of vital significance for microorganisms, containing comprehensive information on metabolic pathways from basic sugar metabolism and amino acid metabolism to complex lipid metabolism and nucleotide metabolism, which is not only the basis for microbial survival and reproduction, but also an important pathway for microorganisms to carry out exchange of substances and energy conversion with the environment [50]. Among them, the energy metabolism pathway is part of the core metabolic network, which is crucial for the survival, adaptation, and functional realization of the organism, for which the tertiary metabolic pathways of energy metabolism were further analyzed (Figure 7b). Apparently, high nitrogen loading promoted most of the pathways in energy metabolism, and nitrogen metabolism, sulfur metabolism, and oxidative phosphorylation increased synchronously with nitrogen loading, which showed a consistent trend with the enhanced nitrogen removal performance of the reactor. This indicates that appropriate nitrogen loading can effectively promote the nitrogen removal of the reactor, while carbon fixation, methane metabolism, and photosynthesis did not show significant changes overall.

## 4. Conclusions

The effect of nitrogen loading on the denitrification performance of the anaerobic ammonia oxidation reactor was explored by controlling the influent substrate concentration. Neither low nor high nitrogen loading was favorable for the enrichment of anaerobic ammonia-oxidizing bacteria, and the relative abundance of anaerobic ammonia-oxidizing bacteria as the dominant species increased from 5.85 ± 1.30% in the P1 stage to 11.43 ± 1.02% in the P3 stage; however, the index of microbial diversity in the reactor decreased from 3.78 ± 0.10 in P1 to 3.63 ± 0.10 in P3. In the inhibition stage of nitrogen loading (P2), the modularity index of the co-occurring network reached the maximum value of 0.563, while in the P4 starvation stage, the modularity index reached the second highest value of 0.545, suggesting that microorganisms respond to the adverse effects of nitrogen-loading fluctuations by forming a modular structure, and the Zi-Pi plots in the P2 and P4 stages showed the highest number of microorganisms as the inter-module connectivity, indicating that microorganisms would respond to nitrogen load fluctuations by enhancing the interspecies connectivity to cope with the effects of nitrogen loading. Changes in nitrogen loading altered the overall metabolic level of microorganisms in the reactor, and in practice, controlling the appropriate substrate concentration helped the enrichment of anaerobic ammonia-oxidizing bacteria, and steadily increasing the nitrogen loading step by step helped to improve the denitrification performance of the reactor.

## Figures and Tables

**Table 1:** Synthetic wastewater composition.

Composition	Concentration
(NH_4_)_2_SO_4_	As request
NaNO_2_	As request
NaHCO_3_	0.5 g/L
KHCO_3_	0.5 g/L
KH_2_PO_4_	0.027 g/L
MgSO_4_·7H_2_O	0.02 g/L
CaCl_2_·2H_2_O	0.136 g/L
Trace element Solution I ^a^	1 mL/L
Trace element Solution II ^b^	1.2 mL/L

^a^ Composition of trace element Solution I: 5 g/L EDTA and 5 g/L FeSO_4_·7H_2_O. ^b^ Composition of trace element Solution II: 5 g/L EDTA, 0.22 g/L NaMoO_4_·2H_2_O, 0.19 g/L NiCl_2_·6H_2_O, 0.25 g/L CuSO_4_·5H_2_O, 0.24 g/L CoCl_2_·6H_2_O, 0.43 g/L ZnSO_4_·7H_2_O, and 0.99 g/L MnCl_2_·4H_2_O.

**Table 2:** Alpha diversity index analysis of bacteria.

Phase	ACE	Shannon	Pielou	Good’s Coverage
P1	267.00 ± 13.61 a	3.78 ± 0.10 a	0.69 ± 0.02 a	1.00 a
P2	258.99 ± 8.71 a	3.71 ± 0.07 ab	0.69 ± 0.01 a	1.00 a
P3	269.63 ± 20.91 a	3.63 ± 0.10 ab	0.66 ± 0.01 a	1.00 a
P4	268.51 ± 10.79 a	3.75 ± 0.05 b	0.69 ± 0.01 b	1.00 a
P5	250.85 ± 17.96 a	3.31 ± 0.15 c	0.61 ± 0.02 c	1.00 a

Different letters indicate significant differences before and after composting.

**Table 3:** The topological features of the co-occurrence network for different sampling phases.

Phase	Nodes	Edges	Positive Links (%)	Negative Links (%)	Average Degree	Clustering Coefficient	Modularity
P1	197	1355	57.05	42.95	6.878	0.507	0.499
P2	177	709	54.30	45.7	4.006	0.480	0.563
P3	154	2850	56.84	43.16	13.014	0.575	0.231
P4	197	943	53.02	46.98	4.787	0.520	0.545
P5	187	1135	52.78	47.22	6.07	0.541	0.473

## Data Availability

The data generated from the study are clearly presented and discussed in the manuscript.
